# Stabilization and Resuscitation of Newborns

**DOI:** 10.3390/children9101492

**Published:** 2022-09-28

**Authors:** Bernhard Schwaberger

**Affiliations:** Division of Neonatology, Department of Pediatrics and Adolescent Medicine, Medical University of Graz, 8036 Graz, Austria; bernhard.schwaberger@medunigraz.at; Tel.: +43-316-385-30018

The majority of newborns do not need medical interventions to manage the neonatal transition after birth. However, every year millions of newborns worldwide require respiratory support immediately after birth, and another considerable number of newborns additionally require extensive resuscitation including chest compressions and drug administration. Despite a significant increase in knowledge and development of enhanced therapy strategies over the past few years, morbidity and mortality caused by failures in neonatal transition remain an important health issue. The purpose of this Special Issue is to support or introduce novel concepts and add information in the area of the “Stabilization and Resuscitation of Newborns”, aiming to improve neonatal care and, as the major objective, to enhance neuro-developmental outcomes.

## 1. Respiratory Support

Most emergency situations in the newborn are respiratory conditions, often during the neonatal transition after birth. Neonatologists are confronted with the challenging task of achieving optimal care for term and preterm newborns which often requires quick and decisive action. The review by Aichhorn et al. [1] describes the neonatologist-performed lung ultrasound (NPLUS) as a point-of-care examination and its use for the stabilization and resuscitation of newborns. NPLUS immediately provides knowledge about the respiratory condition of the patient and helps to rule out pneumothorax, and visualize ventilation or atelectases, effusions, or consolidations in less than one minute. More experienced sonographers may use NPLUS for assessment of tracheal tube placement, diagnosis of congenital malformations, diaphragmatic movement, and pneumomediastinum as well as assessment of laryngeal anatomy. Thereby, NPLUS further reduces exposure to ionizing radiation.

Neonatal tracheal intubation is a life-saving procedure, but adverse events are not uncommon. A small randomized controlled trial by Bruckner et al. [2] evaluated the concept of providing continuous gas flow through the tracheal tube during neonatal intubation to prevent severe desaturation or bradycardia. This novel approach seems to be favorable compared to the standard procedure without increasing the risk for major unexpected adverse events.

Meconium aspiration syndrome remains a major contributor to neonatal morbidity and mortality. The study by Fan et al. [3] suggested that thick meconium in newborns might be associated with poorer outcomes compared with thin meconium based on chart reviews. Additionally, they presented cell survival assays following the incubation of various meconium concentrations with monolayers of certain cell lines, which were consistent with the results obtained from the chart reviews.

Lesneski et al. [4] compared time to reversal of ductal shunting in persistent pulmonary hypertension of the newborn (PPHN) between standard and high oxygen saturation (SpO2) targets during resuscitation and in the post-resuscitation period. In this term lamb model of meconium aspiration syndrome and PPHN, targeting SpO2 at a higher range (95–99%) during resuscitation and in the immediate post-resuscitation period led to a quicker transition to left-to-right shunting across the ductus arteriosus but did not result in a sustained increase in pulmonary blood flow.

Inhaled nitric oxide (iNO) is a common therapy for newborns suffering from PPHN in the neonatal intensive care unit, but the potential effects of its use during stabilization and resuscitation immediately after birth have not been investigated in detail. In their animal study, Lakshminrusimha et al. [5] studied the effects of iNO on oxygenation and pulmonary vascular resistance in preterm lambs with and without PPHN during resuscitation and stabilization at birth. They concluded that iNO at birth is effective for improving oxygenation and reducing pulmonary vascular resistance in both preterm lambs with and without PPHN without increasing inspired oxygen.

In newborns treated with therapeutic hypothermia following perinatal asphyxia, several risk factors are associated with adverse outcomes. In their retrospective study including 71 asphyxiated cooled newborns, Giannakis et al. [6] analyzed the association between ventilation status (mechanical ventilation versus spontaneously breathing newborns) and adverse short-term outcomes. The need for mechanical ventilation was significantly higher in newborns with more severe asphyxia. In ventilated newborns, higher levels of encephalopathy, lower partial pressure of carbon dioxide, and increased oxygen supplementation were associated with adverse short-term outcomes.

## 2. Cardio-Circulatory Support

Cardio-circulatory support after birth may include interventions such as chest compressions, the establishment of vascular access and emergent drug administration (e.g., epinephrine, volume expanders), and–in very rare cases–may also include defibrillation.

While the current neonatal resuscitation guidelines recommend a 3:1 compression to ventilation ratio, it was recently demonstrated that providing continuous chest compressions superimposed with high distending pressure or sustained inflation may reduce the time to return of spontaneous circulation and mortality in both asphyxiated piglets and newborn infants. A review by Kim et al. [7] summarizes the currently available evidence of continuous chest compressions superimposed with sustained inflation.

Drug administration during neonatal resuscitation requires immediate vascular access to the newborn. Recent guidelines recommend the umbilical venous catheter (UVC) as the optimal vascular access during neonatal resuscitation. However, the UVC securement may be challenging and time-consuming. Therefore, our study group [8] introduced a new concept of UVC securement by using a peripheral catheter and a disposable umbilical clamp. This experimental study on umbilical cord remnants was designed to test the feasibility of this concept. It may be a rewarding option for umbilical venous catheterization and securement, but the experimental data need to be confirmed in clinical trials before being introduced into clinical practice.

In case of persistent bradycardia despite effective ventilation and chest compressions, the administration of epinephrine via a UVC is recommended and should be followed by a certain amount of flush volume. The flush may be essential to push epinephrine to the right atrium in the absence of intrinsic cardiac activity during chest compression. Sankaran et al. [9] evaluated the effect of 1 mL versus 2.5 mL flush volumes of normal saline after epinephrine administration via a UVC in a near-term ovine model of perinatal asphyxia-induced cardiac arrest. Three out of seven (43%) and 12/15 (80%) lambs achieved the return of spontaneous circulation after the first dose of epinephrine with 1 mL versus 2.5 mL flush, respectively (*p* = 0.08). From this pilot study, higher flush volume after the first dose of epinephrine may be beneficial during neonatal resuscitation.

An adequate blood volume is important for neonatal resuscitation and stabilization. In their retrospective single-center study, Aboalqez et al. [10] quantified the cumulative iatrogenic blood loss in very low birth weight infants by blood sampling and the necessity of packed red cell transfusions from birth until discharge from the hospital. They concluded that iatrogenic blood loss should be limited to a minimum in the interest of patient blood management.

The case presentation by Mileder et al. [11] is the first reported case of a newborn with perinatal asphyxia, who required postnatal resuscitation and defibrillation due to ventricular fibrillation following epinephrine administration. Based on this case, the authors suggest that health care providers managing neonatal resuscitation should be aware of the possible need for defibrillation, even though it may be very rare. Therefore, they suggest providing a defibrillator with appropriately sized pediatric defibrillation pads in every delivery room, allowing for weight-adapted, gradual titration of the energy level.

## 3. Cardio-Respiratory Monitoring

Cardio-respiratory monitoring may assist health care providers to identify the newborn in need of medical interventions after birth and may be used for evaluating and observing the newborn’s condition at the neonatal intensive care unit. For these purposes, pulse oximetry is routinely used during neonatal care. The review by Pritišanac et al. [12] compared non-invasive arterial oxygen saturation monitoring by pulse oximetry (SpO2) to oxygen saturation measurements from arterial blood samples (SaO2) in preterm and term newborns in terms of fetal hemoglobin (HbF) measurements. They found a considerable SpO2-SaO2 bias and concluded that the influence of HbF on SpO2 readings may result in an overestimation of SpO2 for the lower saturation ranges.

Advanced monitoring of the cardio-circulatory system and/or the brain provides further information during the neonatal transition after birth. Baik-Schneditz et al. [13] combined non-invasive cardiac output (CO) monitoring by electrical velocimetry and cerebral near-infrared spectroscopy in term newborns after cesarean section, in order to analyze the potential influence of CO on cerebral oxygenation during the neonatal transition. In term infants with uncomplicated neonatal transition after cesarean section, they found no correlation of CO and cerebral oxygenation.

Healy et al. [14] described in their in silico study that anthropometric measures as weight and length of the newborn affect non-invasive CO measurements. Therefore, inaccurate estimates or measurements of anthropometric values can lead to clinically relevant differences in CO measurements, which are more pronounced in preterm infants compared to term infants. This should be considered in the design of future clinical trials and in the interpretation of previous studies including non-invasive CO measurements in the newborn.

## 4. COVID-19

The recent coronavirus disease 2019 (COVID-19) pandemic affected all segments of health care including obstetrics and neonatology. Pawar et al. [15] published a case series of 20 newborns with features of a hyperinflammatory syndrome associated with prenatal maternal SARS-CoV-2 infection potentially caused by transplacental transfer of anti-SARS-CoV-2 antibodies. Ninety percent had cardiac involvement with prolonged QTc (corrected QT interval), 2:1 atrioventricular block, cardiogenic shock, or coronary dilatation. Other findings included respiratory failure, fever, feeding intolerance and melena. All infants had elevated inflammatory biomarkers and received immunomodulatory therapy (e.g., steroids and intravenous immunoglobulins). Two infants (10%) died. This condition was named “Neonatal Multisystem Inflammatory Syndrome” (MIS-N) in conformity with the post-infectious immune-mediated condition in children, “Multisystem inflammatory syndrome in children” (MIS-C), usually seen 3–5 weeks after COVID-19.

I believe that this Special Issue of *Children* helps to contribute to consolidating the current body of information with the hopes of enhancing and stimulating further studies on all spectra of “Stabilization and Resuscitation of Newborns”.

## References

[1] Aichhorn L., Küng E., Habrina L., Werther T., Berger A., Urlesberger B., Schwaberger B. (2021). The Role of Lung Ultrasound in the Management of the Critically Ill Neonate—A Narrative Review and Practical Guide. Children.

[2] Bruckner M., Morris N.M., Pichler G., Wolfsberger C.H., Heschl S., Mileder L.P., Schwaberger B., Schmölzer G.M., Urlesberger B. (2021). In Newborn Infants a New Intubation Method May Reduce the Number of Intubation Attempts: A Randomized Pilot Study. Children.

[3] Fan H.-C., Chang F.-W., Pan Y.-R., Yu S.-I., Chang K.-H., Chen C.-M., Liu C.-A. (2021). Approach to the Connection between Meconium Consistency and Adverse Neonatal Outcomes: A Retrospective Clinical Review and Prospective In Vitro Study. Children.

[4] Lesneski A.L., Vali P., Hardie M.E., Lakshminrusimha S., Sankaran D. (2021). Randomized Trial of Oxygen Saturation Targets during and after Resuscitation and Reversal of Ductal Flow in an Ovine Model of Meconium Aspiration and Pulmonary Hypertension. Children.

[5] Lakshminrusimha S., Gugino S.F., Sekar K., Wedgwood S., Koenigsknecht C., Nair J., Mathew B. (2021). Inhaled Nitric Oxide at Birth Reduces Pulmonary Vascular Resistance and Improves Oxygenation in Preterm Lambs. Children.

[6] Giannakis S., Ruhfus M., Markus M., Stein A., Hoehn T., Felderhoff-Mueser U., Sabir H. (2021). Mechanical Ventilation, Partial Pressure of Carbon Dioxide, Increased Fraction of Inspired Oxygen and the Increased Risk for Adverse Short-Term Outcomes in Cooled Asphyxiated Newborns. Children.

[7] Kim S.Y., Shim G.-H., Schmölzer G.M. (2021). Is Chest Compression Superimposed with Sustained Inflation during Cardiopulmonary Resuscitation an Alternative to 3:1 Compression to Ventilation Ratio in Newborn Infants?. Children.

[8] Schwaberger B., Schlatzer C., Freidorfer D., Bruckner M., Wolfsberger C.H., Mileder L.P., Pichler G., Urlesberger B. (2021). The Use of a Disposable Umbilical Clamp to Secure an Umbilical Venous Catheter in Neonatal Emergencies—An Experimental Feasibility Study. Children.

[9] Sankaran D., Vali P., Chandrasekharan P., Chen P., Gugino S.F., Koenigsknecht C., Helman J., Nair J., Mathew B., Rawat M. (2021). Effect of a Larger Flush Volume on Bioavailability and Efficacy of Umbilical Venous Epinephrine during Neonatal Resuscitation in Ovine Asphyxial Arrest. Children.

[10] Aboalqez A., Deindl P., Ebenebe C.U., Singer D., Blohm M.E. (2021). Iatrogenic Blood Loss in Very Low Birth Weight Infants and Transfusion of Packed Red Blood Cells in a Tertiary Care Neonatal Intensive Care Unit. Children.

[11] Mileder L.P., Morris N.M., Kurath-Koller S., Pansy J., Pichler G., Pocivalnik M., Schwaberger B., Burmas A., Urlesberger B. (2021). Successful Postnatal Cardiopulmonary Resuscitation Due to Defibrillation. Children.

[12] Pritišanac E., Urlesberger B., Schwaberger B., Pichler G. (2021). Accuracy of Pulse Oximetry in the Presence of Fetal Hemoglobin—A Systematic Review. Children.

[13] Baik-Schneditz N., Schwaberger B., Mileder L., Höller N., Avian A., Urlesberger B., Pichler G. (2021). Cardiac Output and Cerebral Oxygenation in Term Neonates during Neonatal Transition. Children.

[14] Healy D.B., Dempsey E.M., O’Toole J.M., Schwarz C.E. (2021). In-Silico Evaluation of Anthropomorphic Measurement Variations on Electrical Cardiometry in Neonates. Children.

[15] Pawar R., Gavade V., Patil N., Mali V., Girwalkar A., Tarkasband V., Loya S., Chavan A., Nanivadekar N., Shinde R. (2021). Neonatal Multisystem Inflammatory Syndrome (MIS-N) Associated with Prenatal Maternal SARS-CoV-2: A Case Series. Children.

